# Inferring Short-Range Linkage Information from Sequencing Chromatograms

**DOI:** 10.1371/journal.pone.0081687

**Published:** 2013-12-20

**Authors:** Bastian Beggel, Maria Neumann-Fraune, Rolf Kaiser, Jens Verheyen, Thomas Lengauer

**Affiliations:** 1 Department of Computational Biology and Applied Algorithms, Max Planck Institute for Informatics, Saarbrücken, Germany; 2 Institute of Virology, University of Cologne, Cologne, Germany; 3 Institute of Virology, University of Duisburg-Essen, Essen, Germany; British Columbia Centre for Excellence in HIV/AIDS, Canada

## Abstract

Direct Sanger sequencing of viral genome populations yields multiple ambiguous sequence positions. It is not straightforward to derive linkage information from sequencing chromatograms, which in turn hampers the correct interpretation of the sequence data. We present a method for determining the variants existing in a viral quasispecies in the case of two nearby ambiguous sequence positions by exploiting the effect of sequence context-dependent incorporation of dideoxynucleotides. The computational model was trained on data from sequencing chromatograms of clonal variants and was evaluated on two test sets of *in vitro* mixtures. The approach achieved high accuracies in identifying the mixture components of 97.4% on a test set in which the positions to be analyzed are only one base apart from each other, and of 84.5% on a test set in which the ambiguous positions are separated by three bases. *In silico* experiments suggest two major limitations of our approach in terms of accuracy. First, due to a basic limitation of Sanger sequencing, it is not possible to reliably detect minor variants with a relative frequency of no more than 10%. Second, the model cannot distinguish between mixtures of two or four clonal variants, if one of two sets of linear constraints is fulfilled. Furthermore, the approach requires repetitive sequencing of all variants that might be present in the mixture to be analyzed. Nevertheless, the effectiveness of our method on the two *in vitro* test sets shows that short-range linkage information of two ambiguous sequence positions can be inferred from Sanger sequencing chromatograms without any further assumptions on the mixture composition. Additionally, our model provides new insights into the established and widely used Sanger sequencing technology. The source code of our method is made available at http://bioinf.mpi-inf.mpg.de/publications/beggel/linkageinformation.zip.

## Introduction

**Figure 1:**
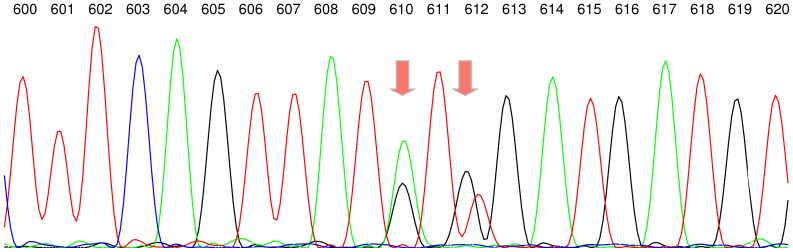
Sequencing chromatogram. The sequencing chromatogram shows two nearby ambiguous sequence positions 610 and 612. At position 610 adenine and guanine are present. At position 612 adenine and thymine are present. Positions are numbered with respect to the *Reverse Transcriptase* of the hepatitis B virus genome. This chromatogram raises the question which of the bases at positions 610 and 612 are present on the same clonal variant.

Direct Sanger sequencing is cheap and widely used but suffers from low sensitivity regarding the detection of minor variants and from the loss of linkage information. The problem of reconstructing the linkage of several ambiguous sequence positions has been addressed only in the setting of diploid genomes so far. Linkage information is of clinical relevance as treatment decisions, for instance for human immunodeficiency virus and hepatitis B patients, are often based on Sanger sequence data of the viral genome [1]–[3]. A particular problem arises when two ambiguous sequence positions are present within the same codon (Figure 1). Then the amino acids expressed cannot be determined precisely due to the lack of linkage information.

Significant attempts have been made to infer linkage information from Sanger sequencing data [4]–[8]. These methods are tailored to genomes that contain a mixture of two different sequences one of which harbors an insertion or deletion. In this case, the chromatogram downstream of the insertion or deletion shows a high number of double peaks as the two genetic variants are superimposed with a phase shift. These methods rely on prior knowledge (reference sequence or set of possible single nucleotide polymorphisms (SNP)) or the availability of both the forward and the reverse chromatogram. Otherwise, such methods can only be applied in situations where the two mixture sequences are sufficiently similar and the analyzed fragment is significantly longer than the insertion or deletion [4]. The method we present here does not rely on diploid genomes that contain a heterozygous insertion or deletion. It is tailored to viral genomes that can have a more complex quasispecies without insertions or deletions. Additionally, we explore a source of information that is encoded in the peak heights of the sequencing chromatograms and has not been considered so far.

Dye-labeled terminator sequencing chromatograms exhibit heterogeneous peak heights that result from different affinities of the polymerase for dideoxynucleotides (chain terminator nucleotides) rather than natural nucleotides during polymerase chain reaction amplification [9]–[11]. The rate of incorporation has been found to also depend on the up- and downstream subsequence [12], [13]. This effect is referred to as sequence context-dependent incorporation of dideoxynucleotides. Methods for estimating the relative frequency of two DNA bases present at a sequence position account for this effect by comparing the observed peak heights to the peak heights of reference chromatograms [13], [14]. Interaction effects between peaks of nearby ambiguous sequence positions are considered to be confounding the interpretation of chromatograms and, therefore, are disregarded or averaged out by these methods. In contrast, we have found that such interactions harbor valuable information for inferring haplotype frequencies. We have developed a probabilistic model that exploits this interaction for the purpose of reconstructing the linkage and thereby also determines the amino acids expressed at two nearby ambiguous sequence positions.

The key assumption of our approach is that the collection of peak heights of a mixture of clonal variants (which are called haplotypes, in the following) is the proportion-weighted mixture of the peak heights of the underlying haplotypes. The underlying physico-chemical reasoning is as follows. Context-dependent incorporation of dideoxynucleotides resulting in context-dependent peak heights occurs at the level of each polymerase molecule that incorporates dideoxynucleotides with an affinity depending on the DNA subsequence it is processing. The observed sequencing chromatogram is the sum of all individual molecular fluorescence impulses. Thus knowing the peak heights of all possible haplotypes present in a mixture, given that these show distinct patterns, renders the determination of the haplotype composition feasible. As context-dependent incorporation of dideoxynucleotides is a local effect the methodology we present here will only infer linkage information over a short genomic region, maybe up to 5 bases. A Gaussian noise model was added to the mixture assumption to account for variation in peak heights. Marginal model likelihoods in combination with the marginals of the model parameters are used for model selection.

## Materials and Methods

In a preliminary study we analyzed a set of sequencing chromatograms from three dilution series published in [14]. This motivated the mixture assumption and provided evidence that sequencing chromatograms contain linkage information and that reconstructing the haplotype composition might be feasible. In order to evaluate our hypothesis we designed and created two *in vitro* test sets, each consisting of four clonal hepatitis B virus (HBV) variants that differ at two nucleotide positions along with two sets of *in vitro* mixtures of the four respective haplotypes.

### Data preparation for the preliminary analysis

The chromatograms of three dilution series published in [14] were obtained from the authors. Each dilution series was created by mixing different pairs of DNA fragments that differ at a single genomic position (single-nucleotide polymorphism). Experimental mixture proportions were 10∶0, 9∶1, 8∶2, 7∶3, 6∶4, 5∶5, 4∶6, 3∶7, 2∶8, 1∶9 and 0∶10. Thus, eleven samples per dilution series were available. Each sample was amplified and sequenced three times.

### *In vitro* experimental setup

The wild-type HBV plasmid vector pCH9-3091 was modified by site-directed mutagenesis. This resulted in a set of six clonal HBV variants (Table 1). The clonal variants 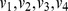 represent all possible haplotypes for the two ambiguous positions 610 and 612 in the *Reverse Transcriptase* shown in Figure 1. Likewise, the haplotypes 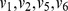 represent all possible haplotypes for two SNPs at positions 610 and 614. Test set 1 (TS1) consists of 29 *in vitro* mixtures of the four haplotypes 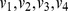 and test set 2 (TS2) consists of 42 *in vitro* mixtures of the four haplotypes 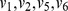. Mixtures were prepared by equimolar mixing. The *in vitro* mixtures were submitted to independent sequencing reactions, each with the same set of oligonucleotides. The compositions of the test sets are available in Table S1.

**Table 1:** Experimental setup.

Haplotype	Position 610	Position 612	Position 614	Comment
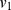	Adenine	Guanine	Adenine	Wild-type
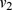	Guanine	Guanine	Adenine	rtM204V
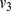	Adenine	Thymine	Adenine	rtM204I
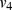	Guanine	Thymine	Adenine	rtM204V
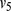	Adenine	Guanine	Thymine	
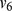	Guanine	Guanine	Thymine	

Six clonal hepatitis B virus genomes (haplotypes) were created by varying three nearby nucleotide positions. Haplotypes 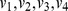 represent all possible combinations of the two variants adenine and guanine present at position 610 and the two variants guanine and thymine present at position 612. These nucleotide variants are clinically relevant as they represent two primary resistance mutations rtM204I and rtM204V. Haplotypes 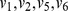 represent all possible combinations of the two variants adenine and guanine present at position 610 and the two variants adenine and thymine present at position 614. Positions are numbered with respect to the *Reverse Transcriptase* of the hepatitis B virus genome.

Clonal variants or mixtures of clonal variants were amplified and sequenced using two distinct established protocols and sequencing machines. TS1 and TS2 were prepared according to [15] and [16], respectively. Sequencing of TS1 was performed on ABI 3130xl using BigDye version 1.1 while TS2 was sequenced on ABI 3730 using BigDye version 3.1. TS2 was prepared during the revision of the manuscript. The use of two different sequencing protocols was due to technological development and not originally intended in the design stage of the study. However, incorporating two technologies enables us to document the robustness of our approach with respect to sequencing protocols.

### *In silico* test set

An *in silico* test set of 1771 samples was created using all possible mixture fractions on a grid with precision 0.05 using haplotype set 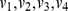. Test chromatograms were computed using the mixture assumption.

### Conditional distribution assumption

The computational approach requires repetitive sequencing of all possible clonal variants in the mixture as training data. Each haplotype has its own characteristic sequencing chromatogram profile because of the effect of context-dependent incorporation of dideoxynucleotides. The chromatogram of a mixture of haplotypes is then the proportion weighted combination of these four haplotype-specific profiles (with some variance). As a preprocessing step all sequencing chromatograms were normalized using a sequence region (called the normalization region) upstream from the region under analysis. Each DNA base (A, T, C, G) requires its own normalization parameter. These four parameters were set such that the average peak height of each base in the normalization region is 1000. Let 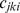 denote the normalized peak heights of the sequencing chromatograms of the four haplotypes with 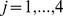 indexing a set of haplotypes (either haplotype set 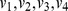 or haplotype set 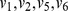), 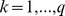 indicating the sequencing replicate and 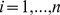 representing all peak heights in a window of about 20 bases around the ambiguous positions. This includes all positions both ambiguous and non-ambiguous. The haplotype-specific profiles 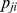 were computed as the median normalized peak heights of the sequencing replicates. 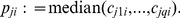
(1)


Given the fractions 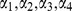 (with 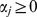 and 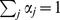) of a mixture of four haplotypes the corresponding mixture profile 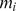 was computed in terms of the weighted sum of the peak heights of the individual haplotypes. Here the weights are the fractions of the haplotypes: 
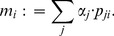
(2)


The likelihood of an observed chromatogram with peak heights 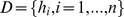 given a mixture profile 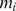 is specified using the conditional distribution assumption: given normalization constants 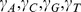 for each DNA base, the mixture components 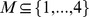 and the corresponding mixture fractions 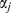, the normalized peak heights 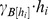 are assumed to be normally distributed with mean equal to the mixture profile peak heights 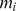 and constant variance 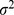. Here 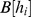 denotes the DNA base of peak height 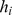.
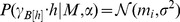
(3)



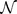
 denotes the likelihood function of the Gaussian distribution and the variance 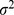 reflects sequencing-dependent variations in peak heights, which were estimated using sequencing replicates of clonal variants.

### Data likelihood

In order to apply equation (3) (to compute the data likelihood 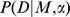) the normalization constants 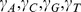 need to be estimated. Therefore, the data 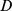 is partitioned into the distinct union of 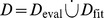. 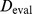 contains all ambiguous and 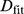 the remaining peak heights. 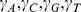 are estimated on 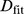 using the maximum likelihood principle given the conditional distribution assumption (3) and the mixture profile 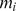. Fitting each normalization constant is an ordinary least-square regression problem for each mixture profile 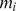. The likelihood of the observation 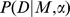 is then computed using equation (4). Thus, we evaluate 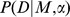 by computing 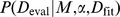. After the normalization constants 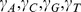 have been estimated using 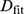 the peak heights of 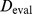 can be assumed to be independent, given the conditional distribution assumption (3). This allows the likelihood function to be factorized.
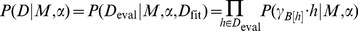
(4)


### Model selection

The primary goal is to determine the haplotypes 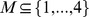 represented by a given chromatogram. Maximum likelihood estimates derived from the conditional distribution assumption overfit and likely produce point estimates indicating that all four haplotypes are present. In order to guard against overfitting regularization is employed by computing the marginal model likelihoods according to equation (5) with uniform priors 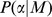 [17].
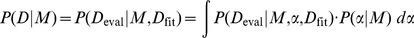
(5)


For the setting with two ambiguous sequence positions seven alternative models need to be considered, which are 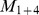, 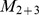, 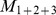, 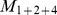, 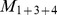, 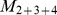 and 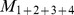. Thus, inferring the haplotype composition is a 7-fold classification problem. The notation 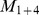 is used to indicate a mixture of haplotypes 1 and 4, 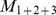 is used to indicate a mixture of haplotypes 1, 2 and 3, etc.. Other subsets of 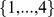, e.g. 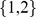, do not exhibit two ambiguous positions in the chromatogram and therefore can be excluded.

Additionally, the modes 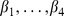 of the posterior marginals of the model parameters 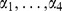 of the full model 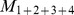 are computed. For each 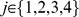 let 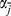 denote all 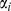 with 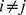.
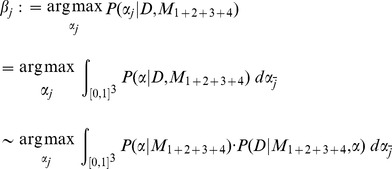
(6)


A model 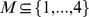 is considered to be inconsistent with 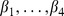 given a certain cutoff value 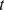 if there exists 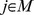 with 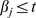 and (in case 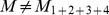) if there is 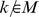 with 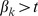. In words, there is a haplotype 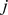 indicated by the model with low 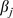 and a haplotype 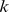 not present in the model with high 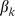, which together indicate a strong contradiction of the model with the parameter marginals. The latter condition can and need only to be fulfilled if the model under consideration is not the full model (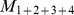). Models that are inconsistent with the modes of the parameter marginals are excluded from the model selection procedure. The cutoff 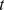 used to evaluate the prediction performance on TS1 was chosen to optimize accuracy on TS2 and vice versa.

The respective integrals (5) and (6) were approximated using a grid of 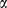 values of precision 0.025. This resulted in 12341 parameter configurations, for which the log-likelihoods were computed and summed up to compute the marginal model likelihoods and the posterior marginal parameter distributions.

### Performance Evaluation

Haplotype reconstruction for two ambiguous sequence positions is a 7-fold classification problem. Prediction performance at the model level is measured in terms of accuracy. Prediction performance is also evaluated at the clonal level. For this purpose, each 7-fold classification problem is interpreted as four 2-fold classification problems defined by the prediction of the presence or the absence of each of the four possible haplotypes. Confidence of predictions is expressed in terms of an uncertainty cutoff. Each prediction is either correct, incorrect or unassigned. A sample remains unassigned if the marginal likelihood of the best model divided by the marginal likelihoods of all other models falls below the uncertainty cutoff.

## Results

We developed a computational model to describe the expected sequencing chromatogram of a mixture of haplotypes. Such a model facilitates the inference of the haplotype composition i.e. to infer the mixture components and fractions. To validate the model two test sets were created. Each test set consists of four clonal HBV variants, which differ at two nearby nucleotide positions, and corresponding test samples of *in vitro* mixtures of the respective haplotypes (Table 1). Further, *in silico* test samples were created to study limitations of our approach.

### Preliminary analysis

**Figure 2:**
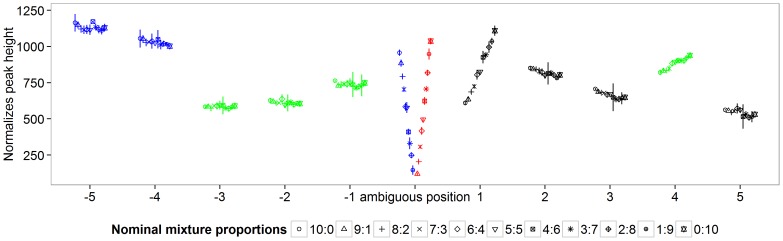
Peak heights of dilution series. The figure shows the median normalized peak heights of the chromatograms of a dilution series sorted by nominal mixture proportion. The normalized peak heights before the ambiguous sequence position are almost identical for all nominal mixture proportions. At the ambiguous sequence position and at up to five bases downstream of the ambiguous sequence position a smooth and apparently linear transition between the peak heights of the samples with nominal mixture proportions 10∶0 and 0∶10 can be observed.

We analyzed chromatograms of a dilution series with nominal mixture proportions 10∶0, 9∶1, 8∶2, 7∶3, 6∶4, 5∶5, 4∶6, 3∶7, 2∶8, 1∶9 and 0∶10. We were interested in changes of peak heights near the ambiguous sequence position and found that the normalized peak heights upstream of the ambiguous sequence position were almost identical for all nominal mixture proportions. At the ambiguous sequence position and at the two to four bases downstream we observed a smooth and almost linear transition of the peak heights between the samples with nominal mixture proportions 10∶0 and 0∶10 (Figure 2). This gave rise to the mixture assumption: the peak heights of a mixture of haplotypes are the proportion-weighted mixtures of the peak heights of the underlying haplotypes. The mixture assumption is common-sense for the ambiguous sequence position in a dilution series. Methods for quantifying base frequencies based on chromatograms rely on this principle [13], [14]. Additionally, we found that this holds for all peak heights. It becomes evident near ambiguous sequence positions where the peak heights of the underlying haplotypes differ due to the effect of sequence context-dependent incorporation of dideoxynucleotides. Finally, Figure 2 shows that there is indeed linkage information in sequencing chromatograms as the peak height at e.g. position 2 depends on the relative frequencies of the bases present at the ambiguous sequence position.

**Figure 3:**
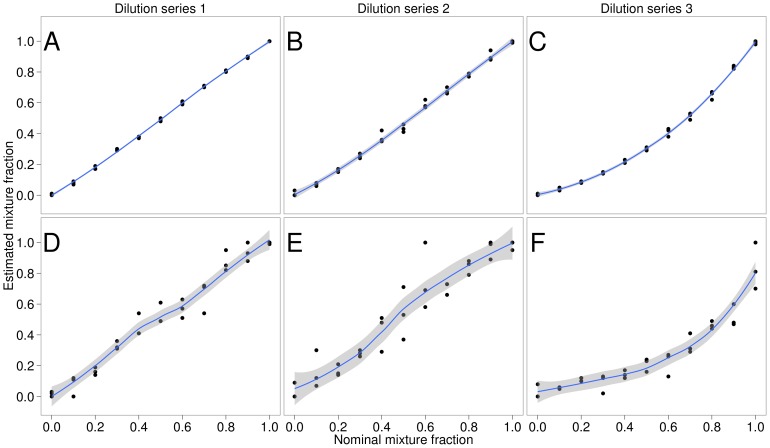
Fraction estimates for dilution series. All subplots show nominal mixture fractions versus estimated mixture fractions for three dilution series. A–C use all peak heights and provide proportions estimates with low error and low variance. D–E ignore the peak heights at the ambiguous positions and try to estimate the mixture proportions based on the unambiguous positions only. The resulting fraction estimates show higher error and higher variance.

The mixture assumption was employed to estimate the mixture fractions of three dilution series. The maximum likelihood estimates for the mixture fractions derived from the chromatograms were close to the nominal mixture fractions for dilutions series 1 and 2 with average absolute errors of 0.01 and 0.03, respectively (Figures 3A and 3B). Dilution series 3 showed a nonlinear relationship between nominal and estimated proportions, resulting in an average absolute error of 0.14 (Figure 3C). This nonlinear relationship is likely caused by varying amplification efficiencies of the different clonal variants. Almost identical results for these data were reported by [14]. Note that the variance in the estimates originates from repetitive amplification and sequencing of the same *in vitro* mixture. The experiment described above was repeated while blanking out the ambiguous positions. Thus, we employed only sequence positions −2, −1, 1 and 2 (Figure 2) to estimate the mixture fractions. This resulted in fraction estimates with higher average errors of 0.05, 0.08, 0.26 and higher variances (Figures 3D–3E). The mixture assumption facilitates the estimation of the fractions of a mixture without using the ambiguous positions. Previous methods that quantify mixture fractions based on sequencing chromatograms only consider ambiguous positions and neglect the information provided by surrounding peaks [13], [14].

We now focus on the problem of haplotype reconstruction based on sequencing chromatograms in the special case of two ambiguous sequence positions. For this problem four possible haplotypes 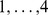 with fractions 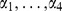 need to be considered. Table 1 rows 1 to 4 lists the four possible haplotypes for the combination of two important HBV drug resistance mutations within the *Reverse Transcriptase* domain. The haplotype reconstruction problem has three degrees of freedom 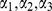 which determine 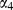 due to 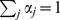. An estimate of the fraction of the first ambiguous position 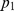 gives rise to 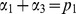 as haplotype 1 and 3 are wild-type at the first ambiguous position. Similarly, an estimate 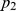 of the fraction of the second ambiguous position provides 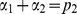. These frequency estimates can be derived from the chromatograms thus reducing the complexity to one degree of freedom. This implies that the peak heights of the ambiguous positions impose strong constraints that limit the space of possible solutions to a straight line in the three-dimensional space of all possible solutions. Within this set of possible solutions we face the problem of model selection. Further evidence from the surrounding peaks or slightly different configurations of the four ambiguous peaks are required to localize the correct solution.

### *In silico* experiment

In order to study elementary properties of our approach we created a set of *in silico* test samples. 1771 artificial chromatograms were created for the set of haplotypes 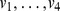 (see Table 1) based on equation (2) using a grid of 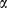 values with grid size 0.05. Test data were generated without adding a noise component. The noise-free setting provides an upper bound on the model performance to be expected on real-world data and simplifies the analysis of model characteristics and limitations. Mixture chromatograms were truncated to nucleotide positions 600 to 620. Positions 610 and 612 were employed to compute the data likelihoods. The value of the standard deviation 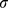 was estimated using the sequencing repetitions of the haplotypes. 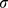 was set to 20.94.

**Figure 4:**
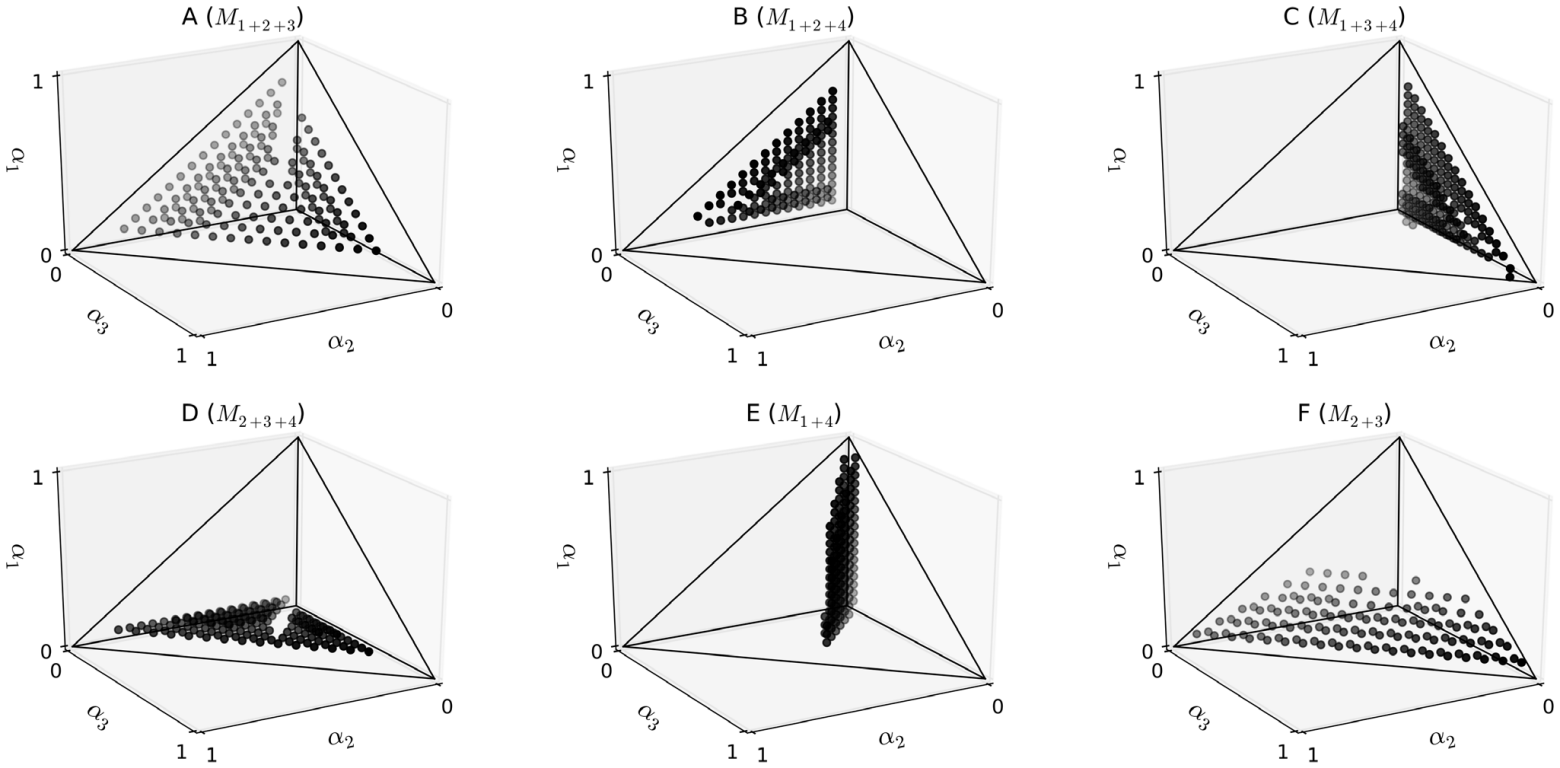
*In silico* prediction results. 1771 *in silico* test chromatograms were created by computing the mixture profiles on a grid of 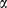 values with precision 0.05. Test chromatograms were classified by the mixture model with 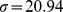. The subplots show all falsely classified samples separately for each falsely predicted label. Six major cases of misclassification can be observed. Subplots A–D show test samples that consist of four haplotypes with at least one haplotype having low frequency. Subplots E and F show test samples that were predicted as mixtures of haplotypes 1 and 4 or of haplotypes 2 and 3, respectively. The data points of subplot E satisfy the linear constraints 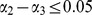, 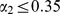 and 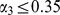. The data points of subplot F satisfy 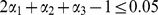, 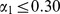 and 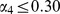.

Only 867 (49%) of the 1771 *in silico* test samples were predicted correctly. Figure 4 visualizes all incorrectly predicted test cases separately for each falsely predicted label. The four surfaces of the simplex correspond to the four 3-mixture models 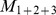, 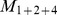, 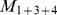 and 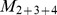. Test samples that lie in the interior of the simplex but close to one of its faces display mixtures of all four haplotypes with one haplotype having low frequency (Figures 4A–4D). These test samples were regularized towards the surface by using the marginal likelihoods for model selection. This reveals a major restriction of the model in terms of sensitivity.

The test set can be divided into two groups: those samples that contain at least one haplotype with a fraction of no more than 10%, and those for which each present haplotype occurs in a fraction higher than 10%. The samples of the first group were predicted correctly in 383 (32.0%) of 1200 cases. The samples of the second group (all present haplotypes exceed a fraction of 10%) were predicted correctly in 484 (85%) of 571 cases.

Figure 4E indicates a second systematic failure: 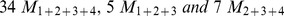 samples were incorrectly predicted as mixtures of haplotypes 1 and 4. All of these samples satisfied the set of linear constraints 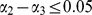, 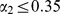 and 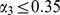. Similarly, 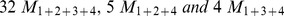 samples were falsely predicted as mixtures of haplotypes 2 and 3 (Figure 4F). These samples satisfied the constraints 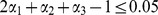, 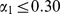 and 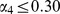. In order to improve prediction performance we analyzed the marginal posterior distributions of the fraction parameters 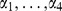. E.g. for all of the seven 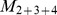 test samples that were misclassified as 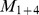 we observed that the modes of posterior marginal distributions of 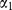 were zero while the modes of 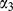 had a mean of 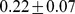. Thus, the modes of the posterior marginals were clearly inconsistent with the model predictions based on the marginal model likelihoods. By checking the consistency of the model predictions with the modes of the posterior marginals using a cutoff-value of 10% (as described in the Methods section) all misclassified 3-mixture test samples could be corrected. Nevertheless, the consistency rule does not improve prediction performance on the 4-mixture test samples. The inability of the model to distinguish between 2-mixture and 4-mixture test samples (if one of two sets of linear constraints is fulfilled) is a major limitation of the model in terms of accuracy.

### *In vitro* validation

**Figure 5:**
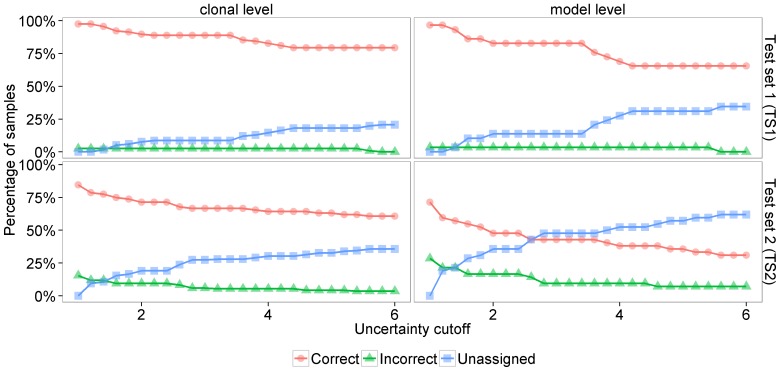
Prediction accuracy on *in vitro* test sets. The figure shows the prediction accuracy on test sets TS1 (subplots A and B) and TS2 (subplots C and D). Prediction accuracy was evaluated both at the clone and at the model level. Each test sample is either predicted correctly, predicted incorrectly or unassigned. The latter happens when the marginal likelihood of the best model divided by the marginal likelihoods of all other models falls below the uncertainty cutoff displayed on the x-axis.

Our hypothesis was that the haplotype composition of arbitrary mixtures can be reconstructed using information from the ambiguous and the surrounding peak heights. In order to test this hypothesis, we created two *in vitro* training sets, each composed of six sequencing repetitions of the underlying haplotypes (Table 1) to compute the individual haplotype profiles and a respective test set of *in vitro* mixtures. Test set 1 (TS1) based on haplotypes 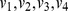 contained 29 mixtures and test set 2 (TS2) based on haplotypes 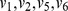 contained 42 mixtures. When model selection was solely based on the marginal model likelihoods prediction accuracy at the model level was 96.6% on TS1 and 47.6% on TS2. By application of the consistency rule as described in the Methods section prediction accuracy improved to 71.4% on TS2 and remained unchanged on TS1. The respective cutoff used to evaluate the prediction performance on TS1 was chosen to optimize accuracy on TS2 and vice versa. A cutoff of 10% was used for both data sets. Note that model selection in our setting is a 7-fold classification problem and the accuracy expected by chance amounts to only 14.3%. Prediction performance was also evaluated at the clonal level. In this evaluation scheme we treated the 7-fold classification problem as four 2-fold classification problems that amounted to the prediction of the presence or the absence of each of the four possible haplotypes. Using this evaluation we could study the accuracy of the model predictions in more detail than when assessing accuracy in terms of 0–1 error at the model level. For instance, the incorrect classification of a 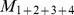 test sample as 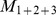 would result in an accuracy of 75% as three of four haplotypes were predicted correctly. Clonal level prediction accuracy was 97.4% for TS1 and 84.5% for TS2. Figure 5 summarizes the prediction performances on TS1 and TS2 both at the model level and at the clonal level. Additionally, we present the results as a function of the prediction confidence expressed by the uncertainty cutoff. E.g. at an uncertainty cutoff of 4.0 only 2.6% (5.4%) of predictions at the clonal level were incorrect, 14.6% (30.4%) were unassigned and 82.8% (64.2%) of predictions were correct on TS1 (respectively TS2).

## Discussion

This article describes the first attempt, to our knowledge, to use peak heights from sequencing chromatograms to infer linkage information about nearby ambiguous positions. We derived a generative model to describe the expected peak heights of a mixture of haplotypes by combining the chromatograms of the underlying haplotypes. The model was motivated by visual inspection of the peak heights of a dilution series. Our model is a generalization of previous methods for estimating the fractions of one ambiguous sequence position [13], [14]. The main difference is that we employ a generative model whose parameters are estimated by probabilistic inference rather than by comparing the observed peak heights to the template peak heights. Previous methods neglected nearby peak heights as they are usually influenced by the ambiguous peaks to be analyzed. In contrast, we exploit this interaction effect to infer linkage information.

The high prediction accuracy of our method on two *in vitro* test sets of 29 and 42 mixture samples showes that sequencing chromatograms do contain linkage information. The prediction accuracy on TS1 was 97.4% at the clonal level and 96.6% at the model level while the accuracy on TS2 was 84.5% at the clonal level and 71.4% at the model level. We found that the marginal distributions of the model parameters 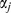 based on the data likelihoods are informative of the mixture composition. Prediction performance at the model level increased from 47.6% to 71.4% on TS2. The Bayesian framework provides confidence values for the predictions in terms of posterior distributions, which can be applied to reduce the number of false predictions at the cost of a higher number of unclassified samples.

The evaluation of the model on a set of 1771 *in silico* test samples revealed several limitations of our approach. First, the model cannot reliably detect minor populations with frequency of no more than 10%. In such cases, the predictions are regularized towards simpler models, which do not contain the low-frequency haplotype. Second, we found that if the mixture fractions 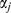 satisfy one of two sets of linear constraints then the mixture is always predicted to be either a mixture of the haplotypes 1 and 4 or to be a mixture of haplotypes 2 and 3. The true mixture components in the former case are 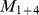, 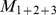, 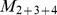 and 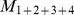 and in the latter case are 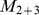, 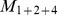, 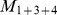 and 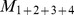. This becomes plausible by looking at the proportion estimates 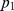 and 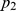 of the two ambiguous positions. If 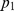 and 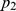 are equal (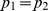), then the observed peak heights can be interpreted to originate from a mixture of haplotypes 1 and 4 with 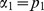 and 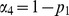. From 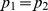 follows 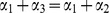 and immediately 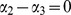. If additionally 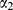 and 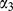 have low frequency (below 35%), we obtain the set of linear constraints that are fulfilled by all *in silico* test cases, which were falsely classified as mixtures of haplotypes 1 and 4 (Figure 4E). The presence of haplotypes 2 and 3 does not become strongly evident by looking at the surrounding peaks due to their relatively low frequency. Similarly, if the frequency estimates at the two ambiguous positions fulfill 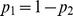, the mixture can be interpreted to contain only the haplotypes 2 and 3 with 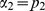 and 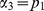. From 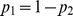 follows 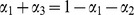 and immediately 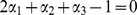, which is the constraint satisfied by all *in silico* test cases that were falsely predicted as a mixture of the haplotypes 2 and 3. This observation is reflected in the *in vitro* test set TS2, in which four samples (9.5%) were incorrectly classified at an uncertainty cutoff of 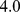. Three samples were 4-mixtures misclassified as 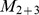 with 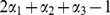 amounted to 0.0, 0.0 and 0.025, respectively. Additionally, one 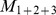 sample was misclassified as 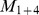 with 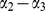 equal to 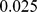.

To summarize, we think that the misclassifications do not result from inaccurate likelihood computations, but rather from the model regularization. Simpler models are preferred, if the peak heights at the ambiguous positions do not imply a more complex model. This is the case, in particular, when one haplotype in a mixture of four haplotypes has low frequency (misclassified as a mixture of three haplotypes), when 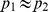 (misclassified as 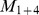) or when 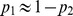 (misclassified as 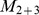).

We evaluated our hypothesis to perform inference on the haplotype compositions for only two sets of sequence positions within the *Reverse Transcriptase* domain of the HBV genome. Nevertheless, peak heights usually display very distinctive patterns and thus we assume the approach will also work in other settings. Two different amplification and sequencing protocols in concert with two different sequencing machines were employed to generate the two test sets. This indicates the robustness of the method with respect to sequencing protocols. The reasoning that the problem is essentially one-dimensional after the mixture fractions are estimated implies that the model very likely cannot successfully be extended to infer the haplotype composition in the presence of more than two nearby ambiguities. Additionally, the locality of the effect of sequence context-dependent incorporation of dideoxynucleotides limits the capacity of the approach to infer phase information of ambiguous positions that are more than a few bases (up to five bases, at most) apart from each other. On test set TS2, in which the two ambiguous positions were three positions apart from each other, prediction accuracy was impaired in comparison to TS1, in which the two ambiguous positions were only separated by a single base.

The advent of next-generation sequencing (NGS) technologies has superseded traditional Sanger sequencing in many applications [18]. NGS technologies offer increased sequencing depth and speed through a high degree of parallelization and miniaturization and allow the detection of minor variants with relative frequencies of as low as 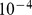 [19]. NGS data also naturally provide linkage information over the whole read length, which may further be extended by the use of paired-end reads [20]. Compared to our approach, NGS technologies are far more sensitive and accurate in determining the haplotype composition of a mixture and can deliver long-range linkage. Nevertheless, traditional Sanger sequencing is cheaper than NGS and NGS will not be accessible in many laboratories for some time to come. Before this background, certain applications, in which the limitations of our approach in terms of sensitivity, accuracy and limited range are acceptable, may not require NGS.

## Conclusions

We have developed and validated an approach to compute the peak heights of a mixture of haplotypes based on the chromatograms of the underlying haplotypes. The model can be used to infer the haplotypes present in a mixture and therefore the short-range linkage for two ambiguous sequence positions. The effect of sequence context-dependent incorporation of dideoxynucleotides — an effect that was previously regarded as detrimental — was employed. The effectiveness of our method shows that short-range linkage information can be inferred from sequencing chromatograms with no further assumptions on the mixture composition. The model also allows the estimation of the fractions of nearby ambiguities and therefore overcomes the limitations of [1]. As a major limitation to its widespread applicability the model requires the sequencing chromatograms of all possible haplotypes in the mixture. The source code of our method can be downloaded at http://bioinf.mpi-inf.mpg.de/publications/beggel/linkageinformation.zip.

## Supporting Information

Table S1 Composition of the *in vitro* test set 1 (TS1) and test set 2 (TS2).
(XLS)
